# Identification of SF-1 and *FOXL2* and Their Effect on Activating P450 Aromatase Transcription *via* Specific Binding to the Promoter Motifs in Sex Reversing *Cheilinus undulatus*

**DOI:** 10.3389/fendo.2022.863360

**Published:** 2022-05-10

**Authors:** Xinxin Ji, Shaoyang Bu, Yi Zhu, Yi Wang, Xin Wen, Feibiao Song, Jian Luo

**Affiliations:** State Key Laboratory of Marine Resorce Utilization in South China Sea, Hainan Aquaculture Breeding Engineering Research Center, Hainan Academician Team Innovation Center, Hainan University, Haikou, China

**Keywords:** promoter, SF-1, *FOXL2*, *cyp19a1a*, *cyp19a1b*, transcriptional regulation

## Abstract

The giant wrasse *Cheilinus undulatus* is a protogynous socially hermaphroditic fish. However, the physiological basis of its sex reversal remains largely unknown. *cyp19* is a key gender-related gene encoding P450 aromatase, which converts androgens to estrogens. *cyp19* transcription regulation is currently unknown in socially sexually reversible fish. We identified *NR5A1* by encoding SF-1, and *FOXL2* from giant wrasse cDNA and *cyp19a1a* and *cyp19a1b* promoter regions were cloned from genomic DNA to determine the function of both genes in *cyp19a1* regulation. Structural analysis showed that SF-1 contained a conserved DNA-binding domain (DBD) and a C-terminal ligand-binding domain (LBD). FOXL2 was comprised of an evolutionarily conserved Forkhead domain. *In vitro* transfection assays showed that SF-1 could upregulate *cyp19a1* promoter activities, but FOXL2 could only enhance *cyp19a1b* promoter transcriptional activity in the HEK293T cell line. Furthermore, HEK293T and COS-7 cell lines showed that co-transfecting the two transcription factors significantly increased *cyp19a1* promoter activity. The −120 to −112 bp (5′-CAAGGGCAC-3′) and −890 to −872 bp (5′-AGAGGAGAACAAGGGGAG-3′) regions of the *cyp19a1a* promoter were the core regulatory elements for SF-1 and FOXL2, respectively, to regulate *cyp19a1b* promoter transcriptional activity. Collectively, these results suggest that both FOXL2 and SF-1 are involved in giant wrasse sex reversal.

## 1 Introduction

In many teleost species, sex fate is not an irreversible deterministic process. Instead, it is actively regulated *via* the suppression or activation of opposing genetic networks, creating the potential for flexibility in sexual phenotype in adulthood (1). Under natural conditions, many fish exhibit sequential hermaphroditism, including female-to-male (protogynous), male-to-female (protandrous), or bidirectional (serial) sex changes. Sex changes involve complex coordinated transformations across multiple biological systems, including behavioral, anatomical, neuroendocrine, and molecular axes. P450 aromatase is a key enzyme in the hormonal steroidogenic pathway (2), playing a switch role in converting androgen to estrogen and is the key enzyme in sex determination and differentiation (3). Estrogens and *cyp19a1a* are critical in controlling ovarian and testicular differentiation in gonochoristic and hermaphroditic fish species (4). Teleosts exhibit intense aromatase activity because of the strong expression of one of the two aromatase genes (aromatase A or *cyp19a1a* and aromatase B or *cyp19a1b*) that arise from a gene duplication event. Interestingly, only radial glial cells (RGC) express aromatase B in adult fish (5).

Fushi-tarazu factor-1 (FTZ-F1) is a member of the orphan nuclear receptor family discovered in *Drosophila*. According to function and expression type, *FTZ-F1* gene is divided into two subgroups, and the liver receptor hormone-1 gene (*LRH-1*) constitutes the nuclear receptor subfamily 5 group A member 2 (*NR5A2)* subgroup, mainly expressed in the liver. Steroidogenic factor-1 (SF-1/adrenal 4 binding protein (Ad4BP)) is part of the *NR5A1* subgroup and is mainly expressed in tissues related to steroid synthesis (6). Therefore, SF-1 is used as a steroid-producing factor to regulate gonadal development and sex determination (7). SF-1 potentially plays a role in transcriptional regulation of P450 aromatase gene (*cyp19a1*) in medaka ovarian follicles (8). SF-1 directly binds to the *cyp19a1* promoter on a conserved binding site to activate *cyp19a1* mRNA transcription in chicken ovaries (9). In mammals, SF-1 knockout male mice showed abnormal and underdeveloped testis structure. Additionally, the expression of *cyp11a* and the steroidogenic acute regulatory protein (*STAR*), two Leydig cell function markers, was impaired, indicating a defect in androgen biosynthesis (10). Collectively, SF-1 could be crucial in sex reversal in giant wrasses by regulating *cyp19* gene transcription.

On the other hand, Forkhead (FH) box (FOX) L2 (FOXL2), another important transcription factor discovered in dermatolysis palpebrarum, is a member of the FOX transcription factor family. In recent decades, *FOXL2* has been recognized as a key gene in ovarian differentiation and egg formation in vertebrates (especially mammals) (11). Many FOXL2 target genes have been discovered, including genes involved in steroid production (such as *STAR*, *cyp17*, and aromatase), inflammation (such as nuclear factor of activated T cells (*NFAT*), prostaglandin-endoperoxide synthase (*PTGS2*), and code dystrophy 2 (*COD2*)), and apoptosis/detoxification (such as manganese superoxide dismutase (*MnSOD*)) (12, 13). *FOXL2* also participates in aromatase transcriptional regulation (14). In Nile tilapia, FOXL2 directly binds to the promoter region of *cyp19a1* through its FH domain to activate *cyp19a1* transcription. FOXL2 can also interact with the ligand-binding domain (LBD) of Ad4BP/SF-1 to enhance Ad4BP/SF-1-mediated *cyp19a1* transcription (15). Recent studies using catfish have shown that *FOXL2* mainly interacts with the cytochrome P450 protein family (16). Similarly, transient transfection of Japanese flounder showed that FOXL2 and cAMP analogs could activate *cyp19a1* transcription *in vitro* (17). These results indicate that *FOXL2* may be crucial in ovarian differentiation in bony fish by regulating aromatase expression and steroid production pathways. Recent studies have shown that FOXL2 can directly bind to SF-1 or act as a nuclear receptor co-modulator to regulate ovarian steroidogenesis and follicular development (15). Therefore, in this study, we presumed that FOXL2 and SF-1 were candidate factors that regulate *cyp19a1* expression in giant wrasses.

As an imminent danger species, the fishing industry has sharply decreased the giant wrasse population (18). However, artificially reproducing this species is limited by the small number of male fish. As a social animal, a colony of giant wrasses allows only one male to be present (19). Indeed, research on sex reversal in giant wrasses is urgent. In the present study, we identified and cloned SF-1 and *FOXL2* cDNA sequences from a giant wrasse and tested both factors in *cyp19a1* transcriptional regulation by utilizing two cell lines (HEK293T and COS-7). Examining *cyp19a1* promoter deletions and mutations confirmed the SF-1 and FOXL2 binding sites in the *cyp19a1* promoter.

## 2 Materials and Methods

### 2.1 The Experimental Fish

A 2-year-old wild and healthy giant wrasse was purchased from the Hainan Qionghai Fishing Port Terminal and Guangzhou Huangsha Fishery Market. The fish was anesthetized in ice, and samples from the brain and gonads were taken and stored in liquid nitrogen for future use. All animal handling procedures were conducted under the Institutional Animal Care and Use Committee of Hainan University.

### 2.2 Cloning of Giant Wrasse SF-1 and *FOXL2* and Expression Vector Construction

RNA was extracted using TRIzol reagent, according to the manufacturer’s instructions. The Prime Script™ RT Reagent Kit with a gDNA Eraser (Perfect Real Time) was used to synthesize cDNA templates. The reverse transcription products were stored at −20°C for later use. cDNA fragments were cloned into PGEM-Teasy plasmids using the SF-1 F1, SF-1 R1, *FOXL2* F1, and *FOXL2* R1 primers to amplify the target fragments. For this study, SF-1 open reading frame (ORF) F1, SF-1 ORF R1, *FOXL2* ORF F1, and *FOXL2* ORF R1 were used to amplify *Hin*dIII, *Xho*I, and *Xho*I and *Eco*RI restriction sites. According to the restriction endonuclease site existing in pCDNA3.1, a suitable enzyme that was the same as the fragment multi-band restriction site was selected to double-digest the pCDNA3.1 vector. The SF-1 and *FOXL2* ORF sequences were ligated into the pCDNA3.1 plasmid, and then the recombinant plasmid was transferred into the DH5α competent cell. Finally, the recombinant plasmid containing the target fragment was screened and identified, and the promoter sequence was obtained using the GenomeWalker method. The cloning method was performed according to the Universal GenomeWalker™ 2.0 User Manual. **Table 1** lists all the primers used for cloning and vector construction.

**Table 1:** Primers used in genes cloning and vector construction.

Name	Sequence
SF-1 F1	AAGCAGTGGTATCAACGC
SF-1 R1	CCCTCACACGCACGCTTGCTT
FOXL2 F1	ATGATGGCCACTTACCAA
FOXL2 R1	TTAAATATCAATCCTCGTGTGTAACG
SF-1 ORF F1	CCGCTCGAGATGTTGGGAGATAAATCTCACG
SF-1 ORF R1	CCCAAGCTTTCACACGCACGCTTGCTT
FOXL2 ORF F1	CCGCTCGAGATGATGGCCACTTACCAAAACC
FOXL2 ORF R1	GGCCTTAAGTTAAATATCAATCCTCGTGTGTAACG
Cyp19a1a-Q-F1	CGGGGTACCAAAAAATGTTTTTGCAGCATTCATT
Cyp19a1a-Q-R1	CCCAAGCTTTGCCACTGAGGTAGCATTTC
Cyp19a1b-Q-F1	CGGGGTACCAGGCAGGAAACACTCACACTC
Cyp19a1b-Q-R1	CCCAAGCTTAAGCCTTCGCCTTACTGGTTG
S-A1A-1255-F	CCCAAGCTTCTCTACAAGCCCTCTAGGAC
S-A1A-111-F	CCCAAGCTTAGGCTGGCATGAATCCAG
S-A1A-21-F	CCCAAGCTTGGTCAGCGGCTCACACTT
S-A1B-1795-F	CCCAAGCTTAAGTGCTTGGACCAAAAG
S-A1B-1534-F	CCCAAGCTTCAGCATCAGCATGTCCTT
S-A1B-1501-F	CCCAAGCTTCGTCGGCTCTTCTTCCAG
S-A1B-864-F	CCCAAGCTTCTTTCTTTGCATCGACA
S-A1B-504-F	CCCAAGCTTAACATTCCTGGAATCC
F-A1B-1097-F	CCCAAGCTTTGTCAAAATCTGTCTGTA
F-A1B-1026-F	CCCAAGCTTGCTAAAATGCAAAGTCCC
F-A1B-871-F	CCCAAGCTTAGAGGAGAACAAGGGGR
Cyp19a1a-MutSF-1#1-F2	CGGGGTACCAAAAAATGTTTTTGCAGCATTCATTCTCTACAAGCCC
Cyp19a1a-MutSF-1#1-R2	GGGCTTGTAGAGAATGAATGCTGCAAAAACATTTTTTGGTACCCCG
Cyp19a1a-MutSF-1#2-F3	CTGTACGCTAGGCTGGCA
Cyp19a1a-MutSF-1#2-R3	TGCCAGCCTAGCGTACAG
Cyp19a1a-MutSF-1#3-F4	TGCATCACCGGTCAGCGG
Cyp19a1a-MutSF-1#3-R4	CCGCTGACCGGTGATGCA
Cyp19a1b-MutSF-1#1-F2	TGGTAACAGATAAGTGCTTGGA
Cyp19a1b-MutSF-1#1-R2	TCCAAGCACTTATCTGTTACCA
Cyp19a1b-MutSF-1#2-F3	GAACCATGCAGCATCA
Cyp19a1b-MutSF-1#2-R3	TGATGCTGCATGGTTC
Cyp19a1b-MutFOXL2#1-F2	GATTCAGGAAGTGCTT
Cyp19a1b-MutFOXL2#1-R2	AAGCACTTCCTGAATC
Cyp19a1b-MutFOXL2#2-F3	AAATGACGTTGTTTGCC
Cyp19a1b-MutFOXL2#2-R3	GAATAAATACAAGGGGAG

### 2.3 Promoters and Sequence Analysis

Pomoter regions of the *cyp19a1a* and *cyp19a1b* were isolated by genome walking. The online software ALGGEN-PROMO (http://alggen.lsi.upc.es/cgi-bin/promo_v3/promo/promoinit.cgi?dirDB=TF_8.3) was used to predict the *cyp19a1a* and *cyp19a1b* transcription binding sites of SF-1 and FOXL2. The giant wrasse’s *NR5A1* and *FOXL2* gene sequences were spliced using DNAMAN software. The ORF was identified using the ORF Finder (https://www.ncbi.nlm.nih.gov/orffinder/). The National Center for Biotechnology Information (NCBI) database (https://www.ncbi.nlm.nih.gov/) was used to identify the coding regions and amino acid sequences. The online software Signal P4.1 was used to predict the signal peptides of *NR5A1* and *FOXL2* genes in the giant wrasse. In addition, an adjacency (neighbor joining (NJ)) phylogenetic tree was constructed based on amino acid sequence alignment using MEGA 5.0. GeneDoc software was used for multiple sequence alignments.

### 2.4 Constructing SF-1 and *FOXL2* Progressive Deletions and Site Mutants in Aromatase *cyp19a1* Promoters

In this study, the *cyp19a1a* and *cyp19a1b* promoters were analyzed using online software, and the SF-1 and FOXL2 binding positions in their sequences were predicted. The promoters were deleted from the analysis. Primers were designed with restriction sites AAGCTT and GGTACC of restriction endonuclease (*Hin*dIII and *Kpn*I). Progressive deletion fragments were constructed using a pGL-4.10 fluorescent expression vector. Based on the effect of transcription factors on the progressive deletion of the promoter, the scope of key transcription binding sites was narrowed, and specific primers were designed to clone the full-length sequence of site-directed mutations by fusion PCR. The ORFs of *NR5A1* and *FOXL2* were ligated using gene-specific primers, restriction endonucleases, and the pCDNA3.1 vector to construct a site-mutant expression vector.

### 2.5 Cell Culture, Transient Transfection, Hormone Treatment, and Luciferase Assays

HEK 293T and COS7 cell lines were grown in Dulbecco’s modified Eagle’s medium (DMEM) supplemented with 10% fetal bovine serum, 2 mM of l-glutamine, 100 U/ml of penicillin, and 0.1 mg/ml of streptomycin in 5% CO_2_ and cultured at 37°C. The confluent cells were seeded in a 24-well plate. The transfection rate was maintained at ~ 90% at the time of transfection. The specific experimental components were as follows: 1) 250 ng of complete 5′ upstream or deletion constructs (normal and mutated) of *cyp19a1a* and *cyp19a1b* promoters cloned into the pGL-4.10 (firefly luciferase) vector and 2) 200 ng of pcDNA3.1 expression plasmid (Invitrogen, Carlsbad, CA, USA) containing ORF cDNAs encoding SF-1 and FOXL2. A 5 ng/well of Renilla luciferase (CMV) vector (Promega, Madison, WI, USA) was used as an internal control. Luciferase assays were performed 48 h after transfection using the Dual-Luciferase Reporter Assay System (Promega). Renilla luciferase activity was used to normalize firefly luciferase activity. Luminescence was measured using a GloMax^®^96 microplate luminometer (Promega). The plasmids used in the transfection experiments were prepared using an Endo Free Plasmid Isolation Kit.

### 2.6 Statistical Analysis

The experimental cell data were set up in four parallels per group. A one-way ANOVA was performed, and the results are expressed as the mean ± SD. Statistical significance was set at *p* < 0.05. * represents a significant difference between the data. SPSS and DPS software was used to perform the statistical analyses and constructed graphs using GraphPad Prism version 5.

## 3 Results

### 3.1 Characterizing SF-1 and *FOXL2* cDNA

**Figure 1:**
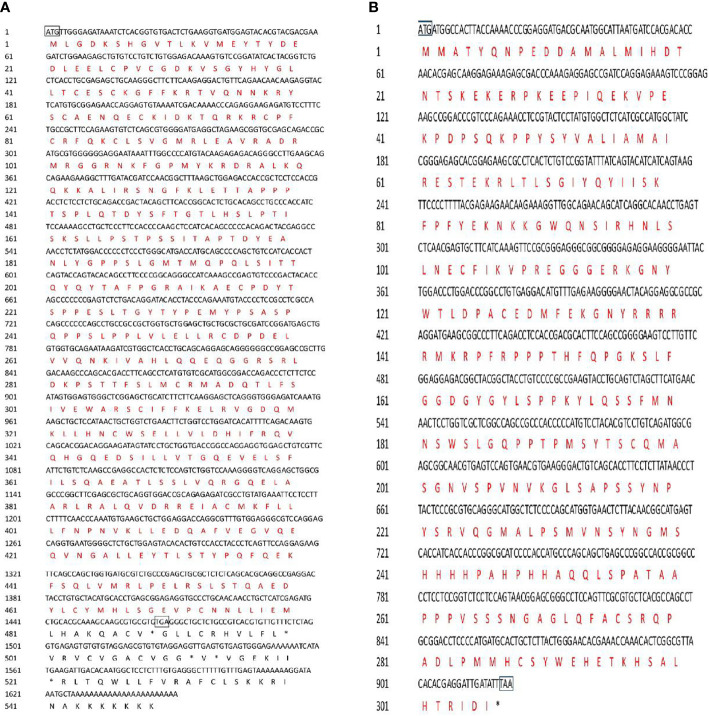
Nucletide and amino acid sequences of *C. undulates SF-1* and *FOXL2*. **(A)** Nucletide and amino acid sequences of *C. undulates SF-1*. The black box marks the start codon ATG and the stop codon TGA. The black typeface is the nucleotide sequence, the red typeface is the amino acid sequence. **(B)** Nucletide and amino acid sequences of *C. undulates FOXL2*. The black box marks the start codon ATG and the stop codon TAA.

The SF-1 and *FOXL2* cDNA sequences were cloned and sequenced. The gene encoding SF-1 had a 1,467-bp ORF encoding 488 amino acid residues (**Figure 1A**). The predicted protein (SF-1) had a molecular weight of 55 kDa and a theoretical isoelectric point (pI) of 7.06. *FOXL2* had a 921-bp ORF encoding 306 amino acid residues (**Figure 1B**). The predicted FOXL2 protein had a molecular weight of 34.59 kDa and a theoretical pI of 9.21.

### 3.2 Homology and Phylogenetic Analysis

**Figure 2:**
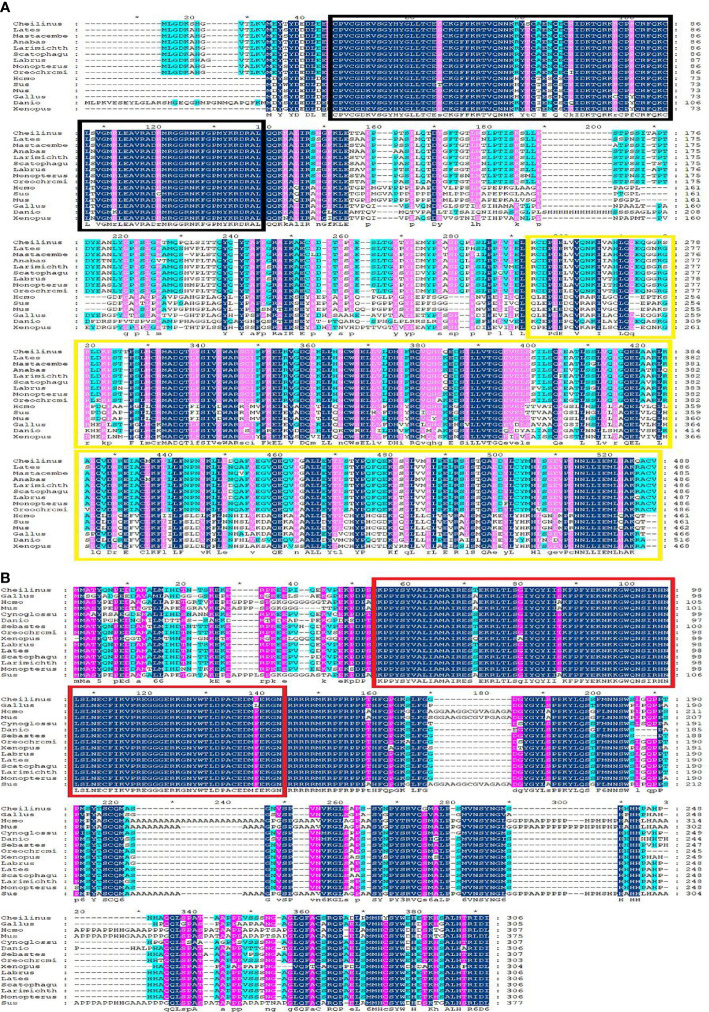
Amino acids sequence alignment of *C. undulates SF-1* and *FOXL2*. **(A)** Amino acids sequence alignment of *C. undulates SF-1*. The black box represents the conserved DNA binding domain (DBD). The yellow box represents the C-terminal ligand binding domain (LBD). **(B)** Amino acids sequence alignment of *C. undulates FOXL2*. The red box represents the conserved Forkhead (FH) domain.

**Figure 3:**
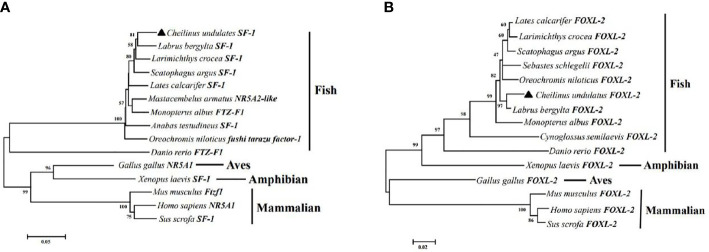
Phylogenetic analysis of *C. undulates* with other vertebrate *SF-1* **(A)** and *FOXL-2* **(B)**. The pylogenetic tree is constructed using the Mega 5.0 adjacency method. Data were resampled with 1,000 bootstrap replicates.

Multiple sequence alignment was conducted to compare the amino acid sequences of SF-1 with those of other vertebrates. It showed that SF-1 was conserved in different aquatic animals (**Figure 2A**). Conserved domain analysis indicated that the SF-1 amino acid sequence contained two domains: a conserved DNA-binding domain (DBD) and a C-terminal LBD. FOXL2 consisted of an evolutionarily conserved FH domain (**Figure 2B**). In addition, a phylogenetic tree was constructed based on the SF-1 amino acid sequences from giant wrasse and 13 other species. As **Figure 3A** shows, SF-1 was most closely related to *Labrus bergylta* SF-1, clustered with *Larimichthys crocea*, and formed a subgroup. SF-1 also showed high similarity to other teleosts and had a relatively long homology with mammalian, avian, and amphibian species. The *FOXL2* homology comparison results were similar to those of SF-1. *FOXL2* showed the closest relationship with *L. bergylta FOXL2. FOXL2* also showed relatively distant homology with mammalian, avian, and amphibian species (**Figure 3B**). In summary, the molecular evolution of SF-1 and *FOXL2* was consistent with species evolution.

### 3.3 Effect of SF-1 and *FOXL2* on *cyp19a1* Promoter Transcriptional Activity

We constructed a luciferase reporter system for *cyp19a1a* and *cyp19a1b* promoters and co-transfected the eukaryotic expression vectors of SF-1 and *FOXL2* into HEK293T and COS7 cell lines to understand how SF-1 and *FOXL2* influence the transcriptional activities of promoters. We used different processing times for different cell lines because the expression time of dual luciferase was unknown after transfection.

**Figure 4:**
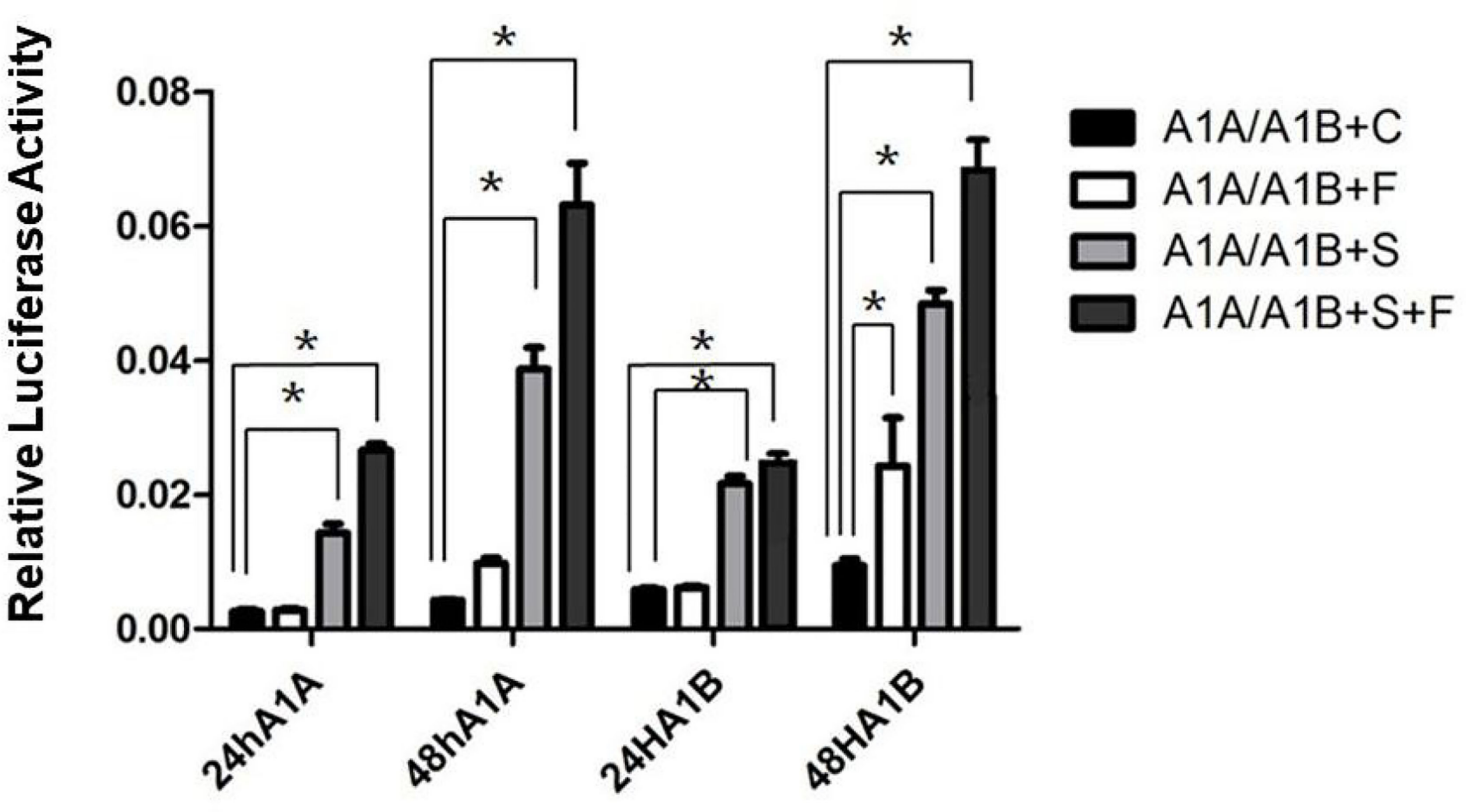
Transcriptional activities of *cyp19a1a* and *cyp19a1b* promoters in HEK293T after 24h and 48h transfection. S meaned that only transcription factor *SF-1* was added, F meaned that only transcription factor *FOXL2* was added. S+F meaned that transcription factors *SF-1* and *FOXL2* were added at the same time. One-way ANOVA was performed on the data and expressed as mean ± standard deviation (n = 4). *There is a significant difference between the data.

**Figure 5:**
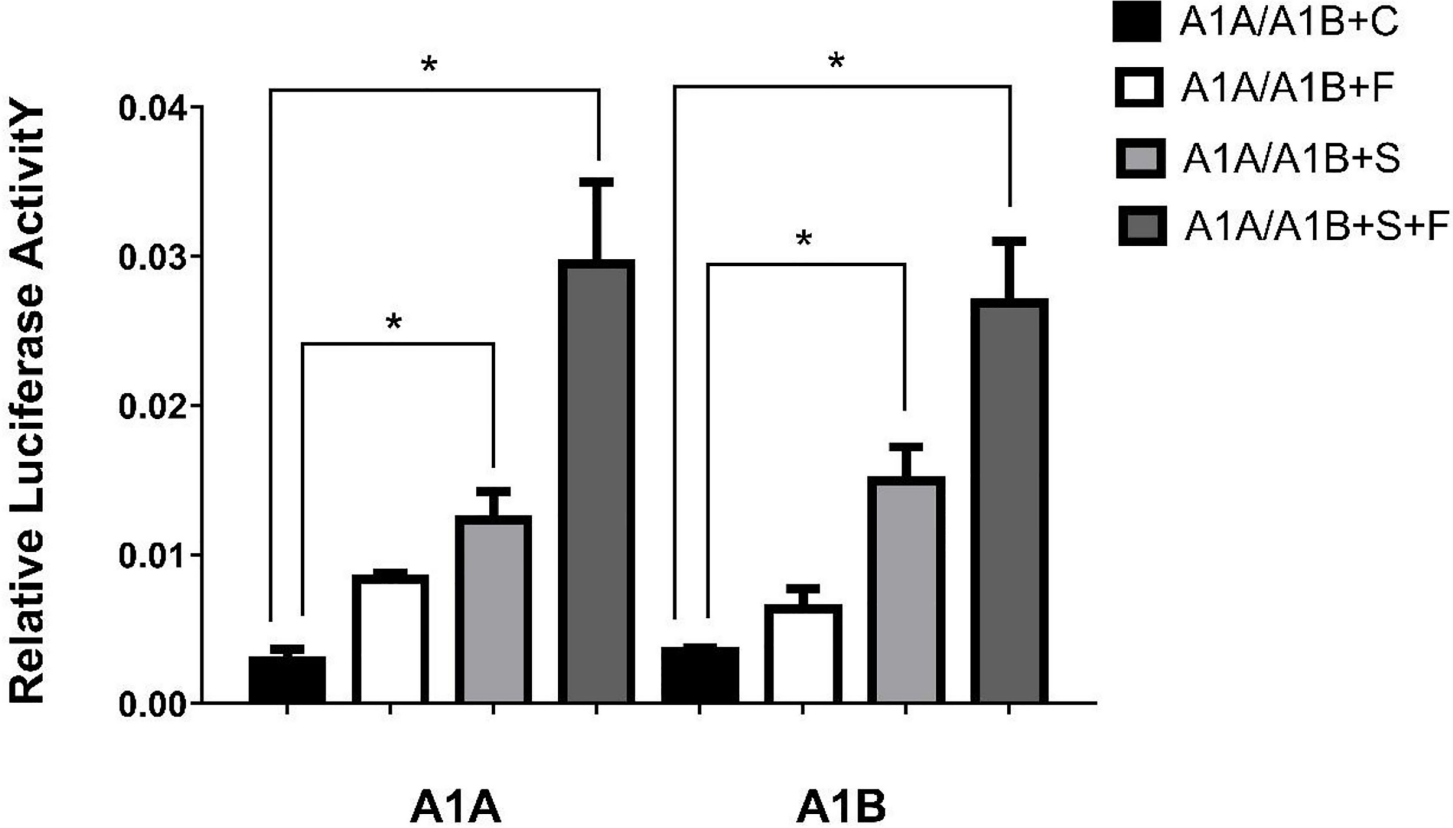
Transcriptional activities of *cyp19a1a* and *cyp19a1b* promoters in COS-7 after 48h transfection. S meaned that only transcription factor *SF-1* was added, F meaned that only transcription factor *FOXL2* was added. S+F meaned that transcription factors *SF-1* and *FOXL2* were added at the same time. One-way ANOVA was performed on the data and expressed as mean ± standard deviation (n = 4). *There is a significant difference between the data.

In the HEK293T cell line, we found that SF-1 significantly upregulated the transcriptional activity of *cyp19a1* promoters 24 and 48 h after transfection, and FOXL2 significantly increased *cyp19a1b* promoter transcriptional activity only after 48 h of transfection (**Figure 4**, *p* < 0.05). At the same time, after SF-1 was transfected into COS-7 cells for 48 h, we observed that the transcriptional activities of *cyp19a1a* and *cyp19a1b* promoters were significantly increased. However, FOXL2 did not increase the transcriptional activity of the *cyp19a1* promoters. Conversely, SF-1 and *FOXL2* co-transfection significantly upregulated *cyp19a1* promoter activity (**Figure 5**, *p* < 0.05). The results of the two cell lines showed that co-transfection of the two transcription factors significantly increased *cyp19a1* promoter activity.

### 3.4 Predicting SF-1 and FOXL2 Transcription Binding Sites

**Figure 6:**
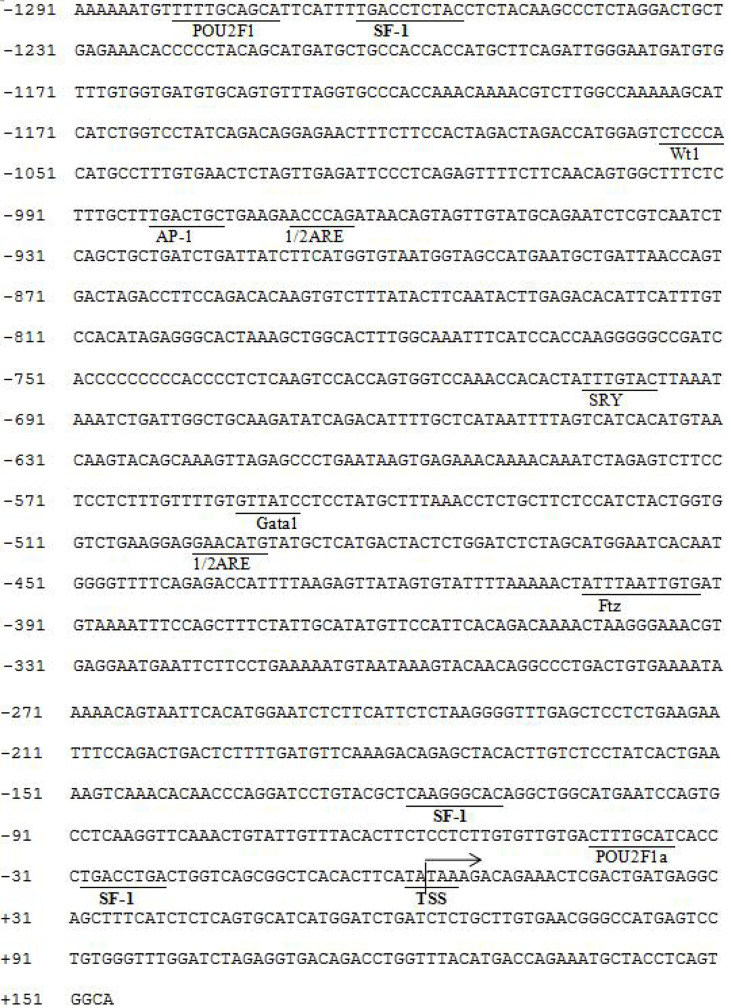
Predicted binding sites of *SF-1* on *cyp19a1a* promoter sequence. The underlined location is the binding site of the predicted transcription binding factor on the initiation. Numbers indicate the distance between the rightmost base in the row and the base of the transcription initiation codo. TSS: Initial site, arrow direction: transcriptional direction.

**Figure 7:**
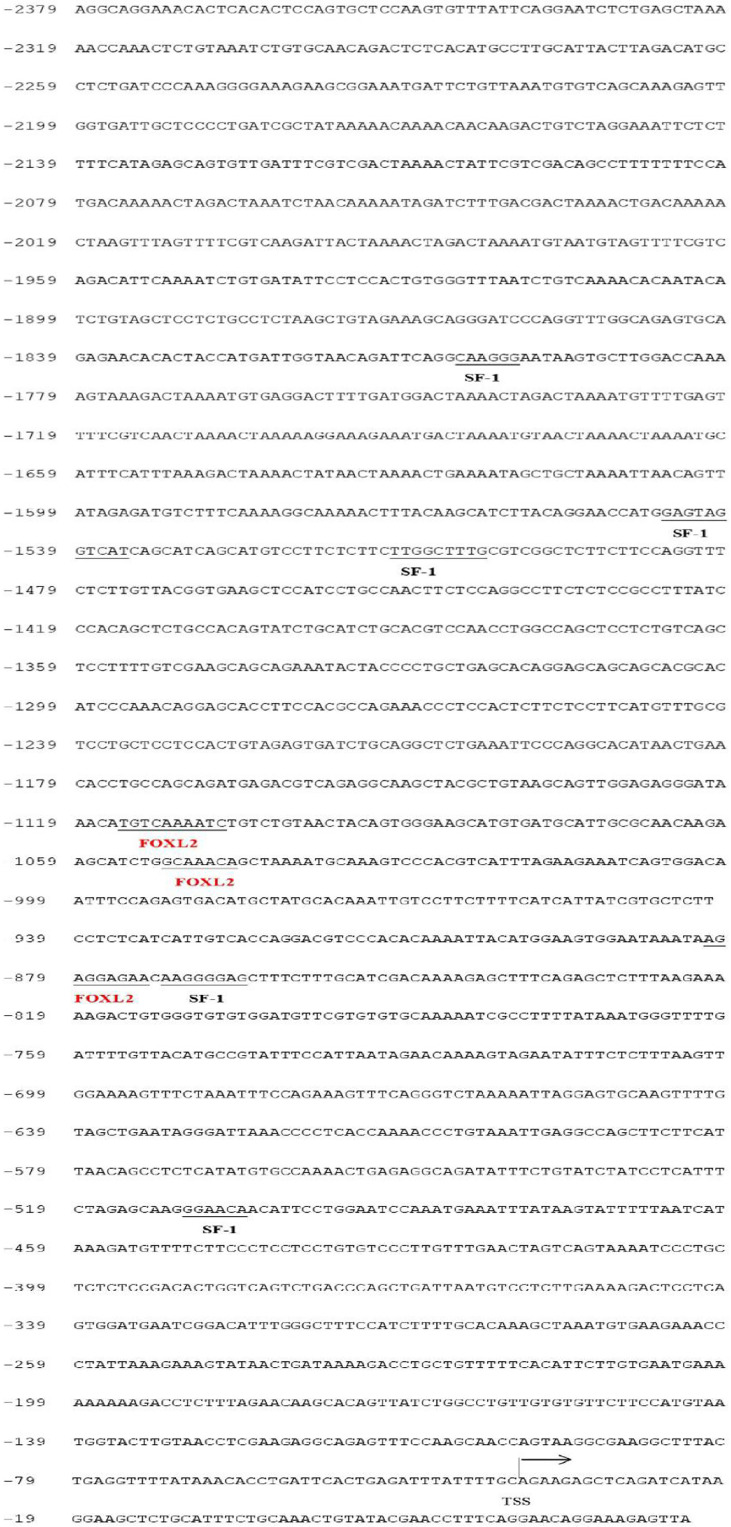
Predicted binding sites of *SF-1* and *FOXL2* on *cyp19a1b* promoter sequence. The underlined location is the binding site of the predicted transcription binding factor on the initiation. Numbers indicate the distance between the rightmost base in the row and the base of the transcription initiation codon. TSS: Initial site, arrow direction: transcriptional direction.

We found that there may be three binding sites for the SF-1 transcription factor and no binding sites for FOXL2 using online software to predict the region of the gonadal aromatase *cyp19a1a* promoter (**Figure 6**). We identified five SF-1 and three FOXL2 transcription binding sites in the brain aromatase *cyp19a1b* promoter (**Figure 7**).

### 3.5 Identifying SF-1 and FOXL2 Transcriptional Binding Sites

We adopted the technology of constructing a progressive deletion of the promoter, fusion PCR, and site-directed mutagenesis to determine the transcription binding sites of the two transcription factors in the aromatase promoters and identify the key elements in transcription regulation. Finally, we identified the key SF-1 and FOXL2 transcription binding sites in the aromatase *cyp19a1* promoters.

#### 3.5.1 The SF-1 Core Binding Site in the Region of Aromatase *cyp19a1* Promoters

**Figure 8:**
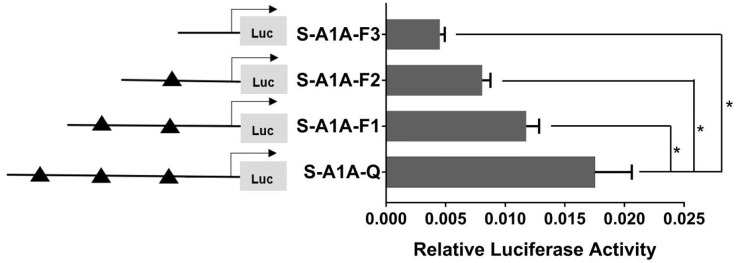
Effect of *SF-1* on the transcriptional activity of the *cyp19a1a* promoter fragment of the progressive deletion site. On the left is a schematic diagram of the promoter deletion and the triangle represents the binding site. The right side is the expression of luciferase in the promoter recombinant vector. S-A1A-F1 (-1291 bp to -1255 bp deletion), S-A1A-F2 (-1291 bp to -111 bp deletion), S-A1A-F3 (-1291 bp to -21 bp deletion). The black triangle represents the *SF-1* binding site. One-way ANOVA was performed on the data and expressed as mean ± standard deviation (n = 4). *There is a significant difference between the data.

**Figure 9:**
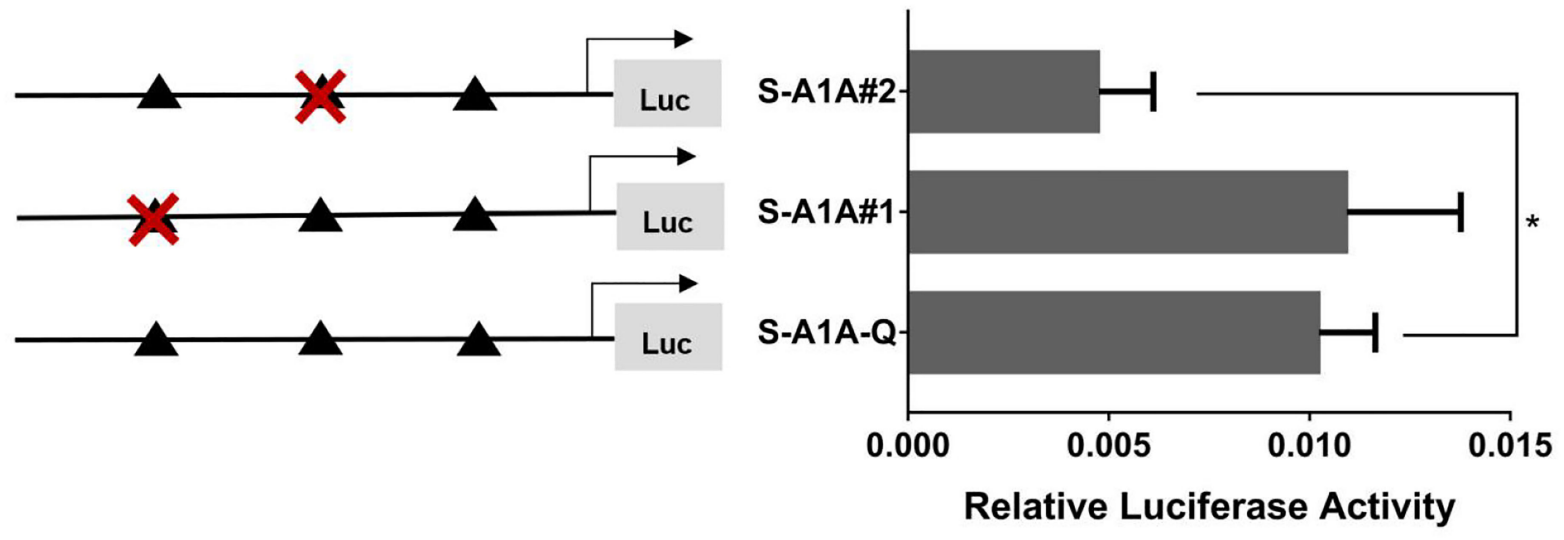
Effect of *SF-1* on the transcriptional activity of the *cyp19a1a* promoter fragment mutated at the transcriptional site. On the left is a schematic diagram of the mutation of the promoter site, and the triangle represents the binding site. The right side is the expression of luciferase in the promoter recombinant vector. The black triangle with a red cross represents the *SF-1* mutation site. One-way ANOVA was performed on the data and expressed as mean ± standard deviation (n = 4). *There is a significant difference between the data.

With the use of the full length of the *cyp19a1* promoter as a reference comparison, deleting −1,291 to −111 bp revealed that the transcriptional activity of the *cyp19a1a* promoter was significantly downregulated (**Figure 8**, *p* < 0.05). We conducted site-directed mutations from −1,265 to −1,256 bp and −119 to −112 bp to determine whether −120 to −112 bp was the key binding site of SF-1 on *cyp19a1a*. We found that mutating −120 to −112 bp significantly reduced *cyp19a1a* promoter activity (**Figure 9**, *p* < 0.05).

**Figure 10:**
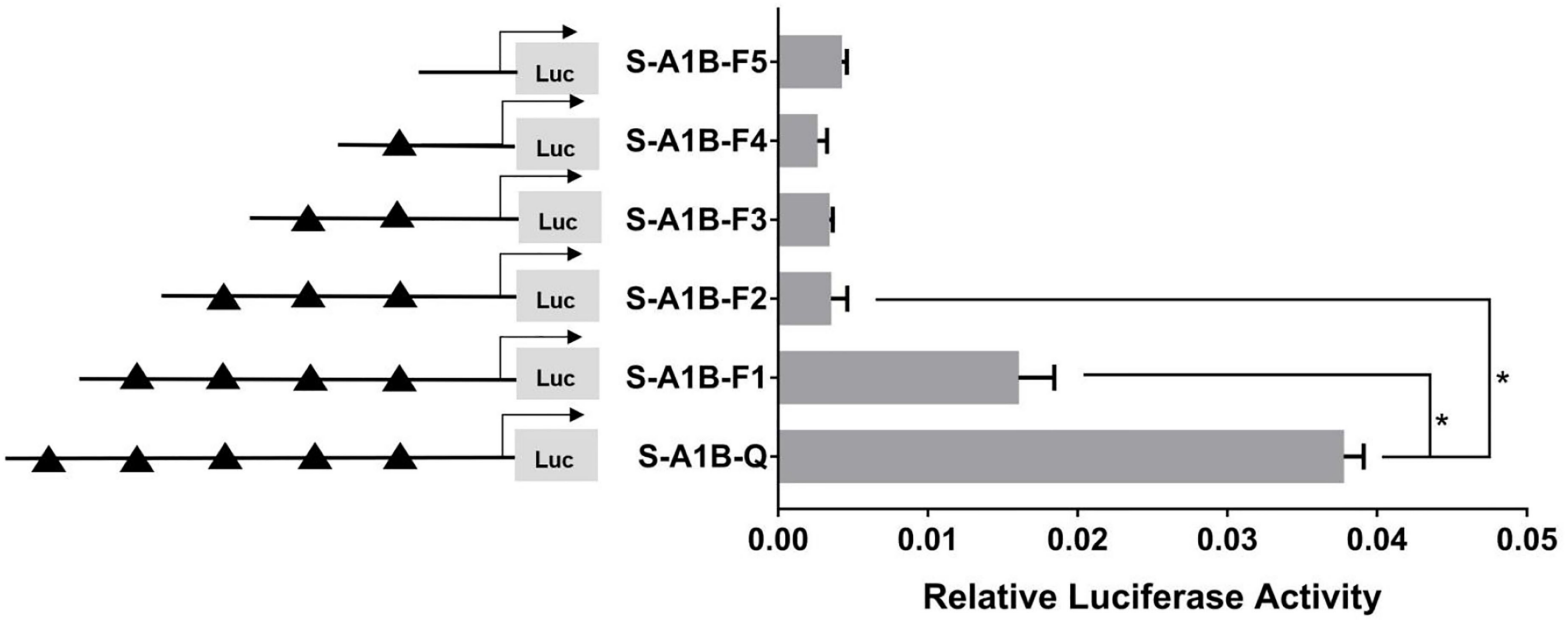
Effect of *SF-1* on the transcriptional activity of the *cyp19a1b* promoter fragment of the progressive deletion site. On the left is a schematic diagram of the promoter deletion and the triangle represents the binding site. The right side is the expression of luciferase in the promoter recombinant vector. S-A1B-F1 (-2379 bp to -1795 bp deletion), S-A1B-F2 (-2379 bp to -1534 bp deletion), S-A1B-F3 (-2379 bp to -1501 bp deletion), S-A1B-F4 (-2379 bp to -864 bp delettion), S-A1B-F5 (2379 bp to -504 bp deletion). The black triangle represents the *SF-1* binding site. One-way ANOVA was performed on the data and expressed as mean ± standard deviation (n = 4). *There is a significant difference between the data.

**Figure 11:**
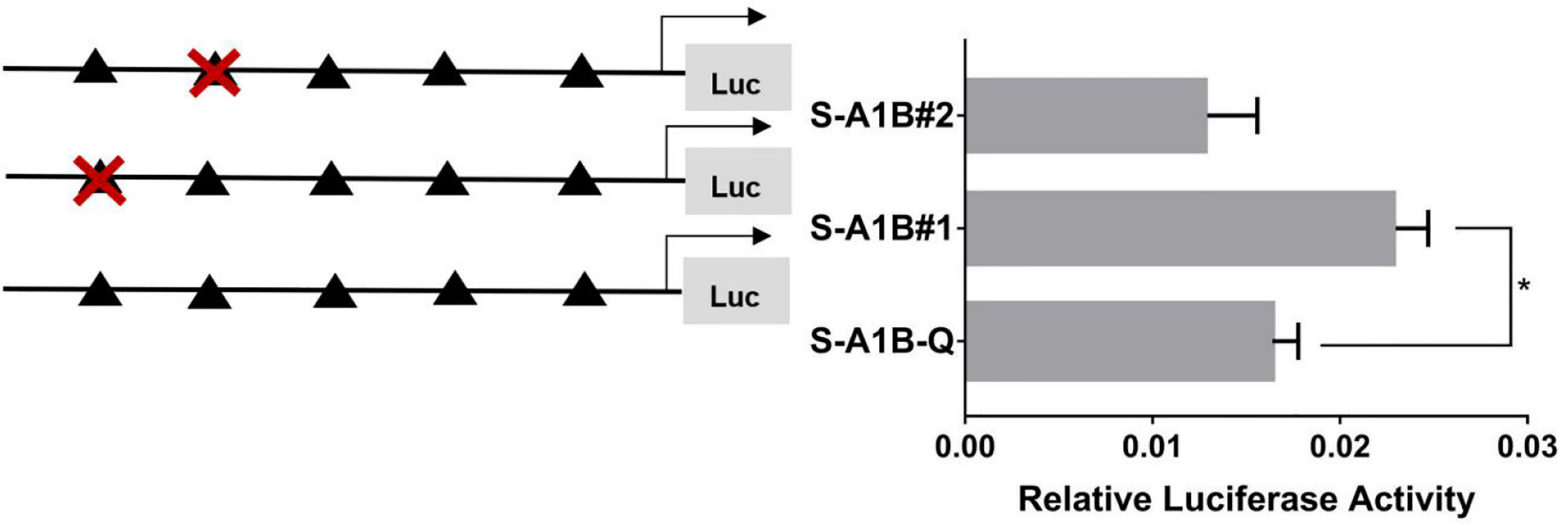
Effect of *SF-1* on the transcriptional activity of the *cyp19a1b* promoter fragment mutated at the transcriptional site. On the left is a schematic diagram of the mutation of the promoter site, and the triangle represents the binding site. The right side is the expression of luciferase in the promoter recombinant vector. The black triangle with a red cross represents the *SF-1* mutation site. One-way ANOVA was performed on the data expressed as mean ± standard deviation (n = 4). *There is a significant difference between the data.

When we deleted the transcription binding sites that predicted SF-1 in the *cyp19a1b* promoter (**Figure 10**, *p* < 0.05), the mutations at these sites did not significantly reduce *cyp19a1b* promoter activity (**Figure 11**, *p* < 0.05). Therefore, we determined that these sites were not the core regulatory sites of SF-1 in the *cyp19a1b* promoter.

In summary, we concluded that −120 to −112 bp (5′-CAAGGGCAC-3′) was the core regulatory element for SF-1 to regulate the transcriptional activity of the *cyp19a1a* promoter.

#### 3.5.2 The FOXL2 Core Binding Site in the Region of Aromatase *cyp19a1* Promoters

**Figure 12:**
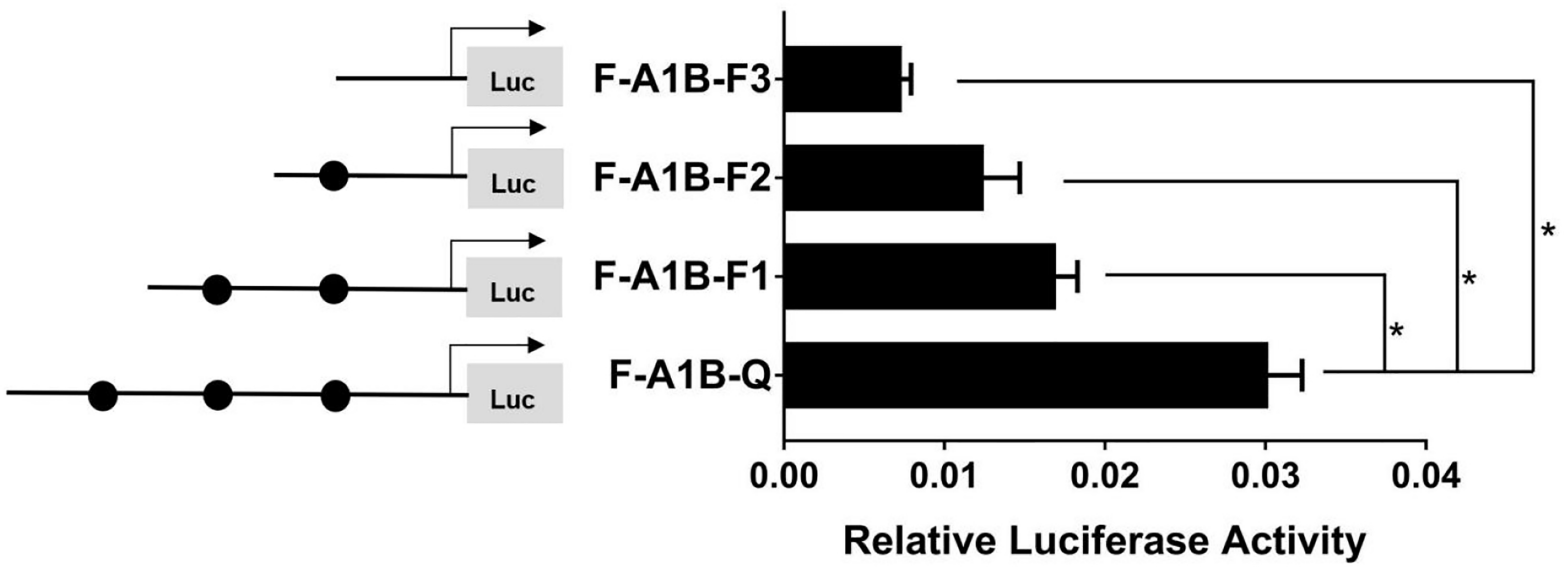
Effect of *FOXL2* on the transcriptional activity of the *cyp19a1b* promoter fragment at the progressive deletion site. On the left is a schematic diagram of the promoter deletion and the circle represents the binding site. The right side is the expression of luciferase in the promoter recombinant vector. F-A1B-F1 (-2379 bp to -1097 bp deletion), F-A1B-F2 (-2379 bp to -1026 bp deletion), F-A1B-F3 (-2379 bp to -871 bp deletion). The black circle represents the *FOXL2* binding site. One-way ANOVA was performed on the data and expressed as mean ± standard deviation (n = 4). *There is a significant difference between the data.

**Figure 13:**
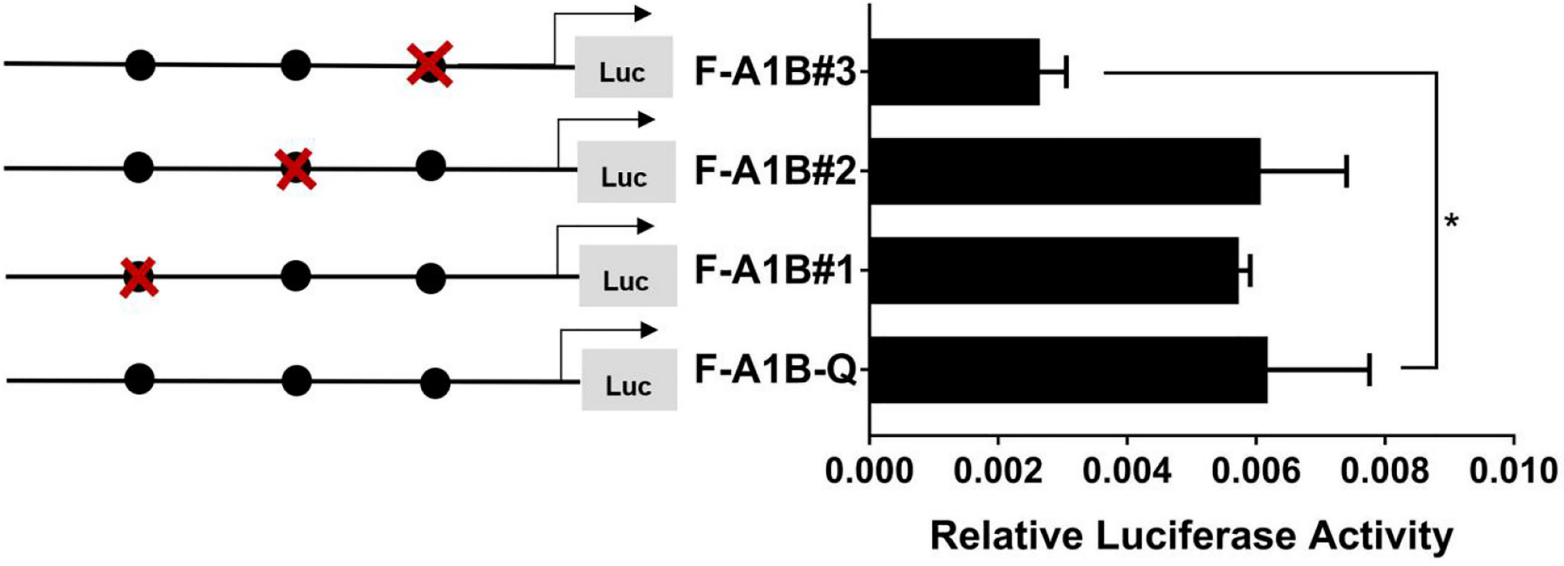
Effect of *FOXL2* on transcriptional activity of the *cyp19a1b* promoter fragment mutated at the transcriptional site. On the left is a schematic diagram of the mutation of the promoter site, and the circle represents the binding site. The right side is the expression of luciferase in the promoter recombinant vector. The black circle with a red cross represents the *FOXL2* mutation site. One-way ANOVA was performed on the data and expressed as mean ± standard deviation (n = 4). *There is a significant difference between the data.

As **Figure 13** shows, we found that after deleting −2,379 to −1,097, −2,379 to −1,026, and −2,379 to −871 bp, the transcription activities decreased significantly from the full length of the promoters (**Figure 12**, *p* < 0.05). Based on the predicted results from −2,379 to −871 bp, we performed site-directed mutations at the following three transcription sites: −1,116 to −1,098, −1,035 to −1,027, and −890 to −872 bp. After mutating −890 to −872 bp, *cyp19a1b* promoter activity decreased significantly (**Figure 13**, *p* < 0.05).

Therefore, we determined that −890 to −872 bp (5′-AGAGGAGAACAAGGGGAG-3′) was the core regulatory element for FOXL2 to regulate the transcriptional activity of the *cyp19a1b* promoter.

## 4 Discussion

*cyp19a1* is the switch enabling androgen to estrogen conversion, affecting gonadal differentiation and sex ratios by regulating the biosynthesis of gonadal steroid hormones (20). We cloned two genes encoding the SF-1 and FOXL2 transcription factors and constructed a phylogenetic tree and conserved domains. Phylogenetic trees showed that the giant wrasse’s SF-1 and *FOXL2* sequences clustered into the teleost group. Similar to a study on *Cyprinus carpio* SF-1, our conserved domain analysis found that the SF-1 amino acid sequence contained two domains: a conserved DBD and a C-terminal LBD (21). Similarly, FOXL2 consisted of an evolutionarily conserved FH domain. This result was the same as that of the conserved domain in the human *FOXL2* gene (22).

### 4.1 SF-1 and FOXL2 Synergistically Upregulate *cyp19a1* Promoter Transcriptional Activities

Evidence supports that NR5A members (FTZ-F1, Ad4BP/SF-1, or LRH-1) and *FOXL2* play a role in the brain. Previous studies have potentiated that NR5A members and *FOXL2* are involved in *cyp19a1a* regulation in some fish and mammals (23–26). Furthermore, the combination of FOXL2 and SF-1 can significantly enhance aromatase *cyp19a1* transcription (27). Similarly, our results showed that co-transfection of *SF-1* and *FOXL2* can enhance the transcriptional activity of the *cyp19a1* promoters, which indicated a synergistic effect between *SF-1* and *FOXL2*. The synergistic effects of SF-1 and FOXL2 have also been verified in other teleosts. For example, in catfish, FTZ-F1/SF-1 and FOXL2 bind to the promoter and upregulate transcription of the aromatase gene in the brain (28). In tilapia, FOXL2 binds to the promoter and interacts with Ad4BP/SF-1 to upregulate aromatase gene transcription in a female-specific manner (15). Similar results were also obtained in ricefield eels, where *NR5A1A* (one of the genes encoding *NR5A* homologs) cooperated with *FOXL2* to upregulate *cyp19a1a* promoter activity (29). However, the synergistic mechanism by which SF-1 and FOXL2 act remains unclear. Studies have shown that SF-1-induced anti-Mullerian hormone (*AMH*) regulation *via* functional FOXL2 occurs through protein–protein interactions between FOXL2 and SF-1 in human granulosa cells (30). In addition, Nagahama et al. reported that FOXL2 could interact with the LBD of *NR5A1* through the FH domain to form a heterodimer in tilapia (15). Researchers in Quebec, Canada, provided a more detailed explanation regarding this synergistic mechanism and found that the zinc finger region of GATA binding protein 4 (GATA-4) mediated GATA-4*/*SF-1 synergy. This synergy is the result of direct protein–protein interactions (31). These results collectively indicate that FOXL2 and SF-1 may affect *cyp19a1* transcriptional regulation through a specific connection or interaction.

### 4.2 SF-1 at the Core Binding Site of the *cyp19a1* Promoter Region

In many bony fish, SF-1 binding sites exist in the promoter region of the gonadal aromatase *cyp19a1* (32–35). In *Oncorhynchus mykiss*, it was found that only −150 to −142 bp and −118 to −110 bp were the core transcription binding sites in the *cyp19a1a* promoter (36). In the orange-spotted grouper, deletion of the region (−246 to −112 bp) containing two conserved FTZ-F1 sites significantly reduced the transcriptional activity of the *cyp19a1a* promoter (37). We hypothesized that −120 to −112 bp were the key transcriptional binding sites of SF-1 in the *cyp19a1a* promoter. Collectively, these results indicated that SF-1 family proteins are common transcriptional regulators of gonad-type *cyp19a1a* genes in fish and provided evidence that they regulate *cyp19a1* expression in vertebrate ovaries. Interestingly, we did not find the core regulatory site of SF-1 in the *cyp19a1b* promoter. From fish to mammals, SF-1 appears to be the common transcription factor of gonad-type *cyp19a1a* but not brain-type *cyp19a1b*. Other studies on aromatase promoters have found that in some fish, including ricefield eel (38), *Epinephelus akaara* (37), zebrafish (34), and *Sparus macrocephalus* (39), only the *cyp19a1a* promoter contains the SF-1 binding site, while the brain-type *cyp19a1b* promoter does not. Therefore, we speculated that *Cheilinus undulatus* may be the same as these fish. Thus, this explanation is reasonable. For example, the SF-1 binding site was found in the mouse brain-type *cyp19a* promoter (40). A previous study found that *NR5A1* knockout mice still contained aromatase in some of their brain cells (41), suggesting that SF-1 may not be necessary for *cyp19a1b* expression.

### 4.3 FOXL2 at the Core Binding Site of the *cyp19a1* Promoter Region

Previous studies have shown that FOXL2 is involved in regulating *cyp19a1* promoter transcription in vertebrates. FOXL2 and SF-1 co-regulate aromatase transcription (15, 27, 28). However, some researchers believe that FOXL2 directly binds to some DNA sites through its FH. Other studies have shown that FOXL2 binds to the half-site sequence of nuclear receptors, namely, the SF-1 response element (SFRE). The SFRE acts on the aromatase promoter (42). Our experiment found that deleting or mutating −890 to −872 bp significantly reduced *cyp19a1b* promoter activity. Therefore, we speculated that −890 to −872 bp is the core element for FOXL2 to regulate *cyp19a1b* transcriptional activity. Our results are consistent with those reported for tilapia. A mutation in the FOXL2 binding site (−545 to −538 bp) in the *cyp19a1b* promoter of tilapia significantly reduced the activity of the promoter (15). Similarly, when a FOXL2 binding site in the promoter of catfish *cyp19a1b* was mutated from −977 to −954 bp, *cyp19a1b* transcriptional activity was significantly downregulated, indicating that this region was the core element of FOXL2 to regulate *cyp19a1b* transcriptional activity (28). These studies all point to the possible involvement of FOXL2 in regulating *cyp19a1b* promoter transcription.

In conclusion, this study found that SF-1 and FOXL2 can regulate *cyp19a1a* and *cyp19a1b* promoters, respectively. SF-1 and FOXL2 contain key binding elements in the *cyp19a1a* and c*yp19a1b* promoters, respectively. The combination of SF-1 and FOXL2 enhanced aromatase promoter activity in giant wrasse. This study lays a theoretical foundation for identifying the aromatase gene transcriptional regulatory pathway in giant wrasse.

## Data Availability Statement

The raw data supporting the conclusions of this article will be made available by the authors on reasonable request.

## Ethics Statement

The animal study was reviewed and approved by the Institutional Animal Care and Use Committee of Hainan University. Written informed consent was obtained from the owners for the participation of their animals in this study.

## Author Contributions

YZ, YW and XJ was the main performer of the experiment. XJ and YZ completed the manuscript, the revision of the article and result graphing. SB analyzed the data and submited the article. XW, FS provided suggestions for writing articles. JL designed all the experiments, provided the required facilities and guidance. All authors read the final article and approved its submission.

## Funding

This work was supported by the National Natural Science Foundation (Grant No. 3166130111).

## Conflict of Interest

The authors declare that the research was conducted in the absence of any commercial or financial relationships that could be construed as a potential conflict of interest.

## Publisher’s Note

All claims expressed in this article are solely those of the authors and do not necessarily represent those of their affiliated organizations, or those of the publisher, the editors and the reviewers. Any product that may be evaluated in this article, or claim that may be made by its manufacturer, is not guaranteed or endorsed by the publisher.
